# Emergence and Potential Extinction of Genetic Lineages of Human Metapneumovirus between 2005 and 2021

**DOI:** 10.1128/mbio.02280-22

**Published:** 2022-12-12

**Authors:** Kevin Groen, Stefan van Nieuwkoop, Adam Meijer, Bas van der Veer, Jeroen J. A. van Kampen, Pieter L. Fraaij, Ron A. M. Fouchier, Bernadette G. van den Hoogen

**Affiliations:** a Department of Viroscience, Erasmus MC, Rotterdam, the Netherlands; b Center for Infectious Disease Control, National Institute for Public Health and the Environment, Bilthoven, the Netherlands; c Pediatric Infectious Diseases & Immunology, Erasmus MC-Sophia Children’s Hospital, Rotterdam, the Netherlands; Icahn School of Medicine at Mount Sinai

**Keywords:** human metapneumovirus, molecular evolution, next-generation sequencing, Nanopore technology, classification, phylogeny, duplication in G

## Abstract

Human metapneumovirus (HMPV) is one of the leading causes of respiratory illness (RI), primarily in infants. Worldwide, two genetic lineages (A and B) of HMPV are circulating that are antigenically distinct and can each be further divided into genetic sublineages. Surveillance combined with large-scale whole-genome sequencing studies of HMPV are scarce but would help to identify viral evolutionary dynamics. Here, we analyzed 130 whole HMPV genome sequences obtained from samples collected from individuals hospitalized with RI and partial fusion (*n* = 144) and attachment (*n* = 123) protein gene sequences obtained from samples collected from patients with RI visiting general practitioners between 2005 and 2021 in the Netherlands. Phylogenetic analyses demonstrated that HMPV continued to group in the four sublineages described in 2004 (A1, A2, B1, and B2). However, one sublineage (A1) was no longer detected in the Netherlands after 2006, while the others continued to evolve. No differences were observed in dominant (sub)lineages between samples obtained from patients with RI being hospitalized and those consulting general practitioners. In both populations, viruses of lineage A2 carrying a 180-nucleotide or 111-nucleotide duplication in the attachment protein gene became the most frequently detected genotypes. In the past, different names for the newly energing lineages have been proposed, demonstrating the need for a consistent naming convention. Here, criteria are proposed for the designation of new genetic lineages to aid in moving toward a systematic HMPV classification.

## INTRODUCTION

Human metapneumovirus (HMPV) is a nonsegmented negative-strand RNA virus belonging to the *Pneumoviridae* family. Globally, HMPV is responsible for 5 to 15% of respiratory infections and is one of the leading causative agents of respiratory illness in young children, only second to respiratory syncytial virus (RSV) (1–8). Clinical symptoms caused by HMPV range from mild respiratory illness to bronchiolitis and pneumonia, similar to those caused by RSV (reviewed in reference 9).

HMPV has a genome of approximately 13.3 kb in size and contains 8 genes, which encode 9 proteins. This includes three surface proteins, the short hydrophobic protein (SH), the attachment glycoprotein (G), and the fusion protein (F). These surface proteins can be used for virus genotyping, also in relation to antigenic variation of circulating HMPV (10, 11). In 2004, it was reported that HMPV isolates clustered in two main genetic lineages that are also antigenically distinct (A and B) and that each diverged in two sublineages (A1 and A2 and B1 and B2) (10). In 2006, a further split of sublineage A2 into A2a and A2b was reported (12). Although the presence of a third lineage (A2c) was reported, another study suggested that the A2b lineage should be further divided in A2b1 and A2b2 and that A2b2 can be mistaken for lineage A2c (13, 14). There were also suggestions that the B2 lineage should be further divided into lineages B2a and B2b, although a consensus on this matter has not been achieved (15). Recently, circulation of lineage A2 viruses carrying a 180-nucleotide (nt) or 111-nt duplication in the G gene was reported for the first time in Japan in 2014 and 2017, respectively (16, 17). In the following years, these viruses were also detected in Spain, Vietnam, and China (16–18). One of these genotypes, with a 111-nt duplication, became the dominant circulating genotype in Yokohama city in Japan (16–18).

Studies on HMPV evolution have focused mainly on the genetic variation of the F and G genes (10, 11, 13, 16, 17, 19–21). However, whole-genome sequencing of HMPV might provide insights into virus evolution that will be missed by analyzing (partial) F and G sequences. Recent genetic changes, such as sequence duplications in the HMPV G gene, highlight the importance of monitoring HMPV evolutionary dynamics, but partial genome sequencing will miss any evolutionary events that occur in genes other than F and G. With next-generation sequencing (NGS) platforms rapidly improving, obtaining viral whole-genome sequences has simplified considerably (22). Recent studies reported assays for HMPV whole-genome sequencing using Illumina sequencing, Ion Torrent, or Nanopore technology (23–26). We recently introduced an HMPV whole-genome sequencing method using MinION Nanopore technology (27). In this study, using this method, whole-genome sequences were generated from 130 HMPV strains obtained from samples collected from hospitalized individuals suffering from respiratory tract infections (RTIs) between 2005 and 2021. Additionally, partial F and G gene sequences were analyzed from 144 viruses obtained from patients with an RTI that visited general practitioners (GPs) between 2005 and 2021 to compare circulation of viruses between the hospitalized patients and patients consulting general practitioners. Phylogenetic analyses were performed to study recent evolutionary patterns and used to propose a robust HMPV classification system for emerging virus lineages.

## RESULTS

### Phylogenetic analyses of HMPV whole-genome sequences.

**FIG 1:**
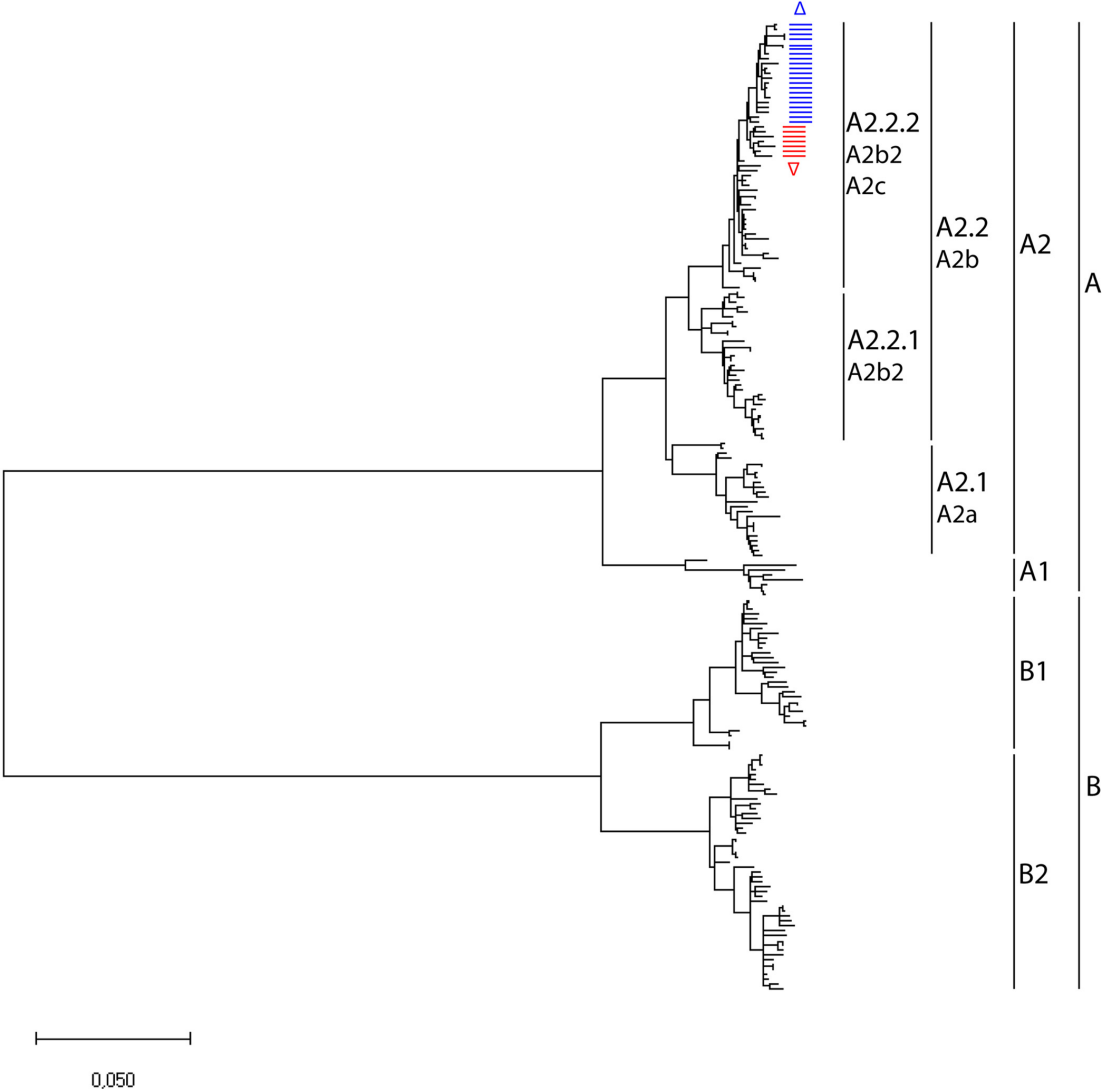
Maximum likelihood phylogenetic tree based on 199 whole HMPV genome sequences. The tree was reconstructed using the Maximimum-Likelihood (GTR+G+I) substitution model with 1,000 bootstraps. The tree with bootstrap values and virus names is shown in Fig. S1 in the supplemental material. Markers on the right indicate the main virus lineages A and B, which could be divided in 6 sublineages (A1, A2.1, A2.2.1, A2.2.2, B1, and B2). Lineage A2.2.2 viruses with a 180-nt duplication (▽, red line) or a 111-nt duplication (△, blue line) in the G gene are highlighted. Different names that have been used for each lineage are shown.

Full-length genome sequences were generated for 130 viruses obtained from hospitalized individuals between 2005 and 2021 and for 13 additional viruses from earlier years (1993 to 2004) that were also not sequenced previously. Phylogenetic analysis of these 143 whole-genome sequences, in combination with all 56 whole-genome sequences available from GenBank (total of *n* = 199), demonstrated clustering of the viruses in the two main genetic lineages A and B (Fig. 1; Fig. S1 and Table S1 in the supplemental material). Within these lineages, separation into the four previously described sublineages A1, A2, B1, and B2 was still observed. Additional subdivisions within lineage A2 were clearly possible, as suggested before (12, 13), but clear criteria for such novel lineage designations are needed. Moreover, different names for the newly emerging lineages have been used in the past, demonstrating the need for a consistent naming convention. For simplicity, lineages from here forward are referred to as A1, A2, A2.1, A2.2, A2.2.1, A2.2.2, B1, and B2. Hereby, A and B are used to distinguish between different phenotypes, and phylogenetic clusters are defined numerically.

10.1128/mbio.02280-22.1 FIG S1 Maximum likelihood phylogenetic tree based on 143 whole HMPV genome sequences obtained in this study combined with 56 from GenBank (total *n* = 199). The sequence set is the same as shown in Fig. 1. The tree was reconstructed using the GTR +G +I substitution model with 1,000 bootstraps. Markers on the right indicate virus lineages and sublineages. Download FIG S1, TIF file, 2.3 MB. Copyright © 2022 Groen et al. 2022 Groen et al. https://creativecommons.org/licenses/by/4.0/ This content is distributed under the terms of the Creative Commons Attribution 4.0 International license.

10.1128/mbio.02280-22.1 TABLE S1 Accession numbers of viruses used as reference for phylogenetic analysis of samples obtained from hospitalized individuals (Fig. 1). Download TABLE S1, PDF file, 0.1 MB. Copyright © 2022 Groen et al. 2022 Groen et al. https://creativecommons.org/licenses/by/4.0/ This content is distributed under the terms of the Creative Commons Attribution 4.0 International license.

In this sample set, viruses with a duplication in the G gene were detected for the first time in 2015 (Table 1). Viruses containing either a 111-nucleotide or a 180-nucleotide duplication in the G gene clustered within the A2.2.2 lineage in two smaller clusters (Fig. 1). The most recent virus with a 180-nucleotide duplication in the G gene was detected in 2018, while from 2019 onward, only viruses with a 111-nucleotide duplication in the G gene were detected. After 2018, no more lineage A2.2.2 viruses without a duplication in the G gene were detected.

**TABLE 1:** Increase in incidence of viruses carrying 180- and 111-nucleotide duplications in the G gene from samples obtained from hospitalized patients within lineage A2.2.2 between 2014 and 2021

Yr	180-nt duplication^*a*^	111-nt duplication^*a*^	No duplication^*a*^	Total^*a*^
2014	–	–	5 (100%)	5
2015	–	2 (29%)	5 (71%)	7
2016	3 (38%)	2 (25%)	3 (38%)	8
2017	2 (29%)	3 (43%)	2 (29%)	7
2018	1 (50%)	1 (50%)	–	2
2019	–	2 (100%)	–	2
2020	–	–	–	–
2021	–	4 (100%)	–	4

a –, no data.

### Comparison of virus clustering based on whole-genome, full-length F, or partial F gene sequences.

**FIG 2:**
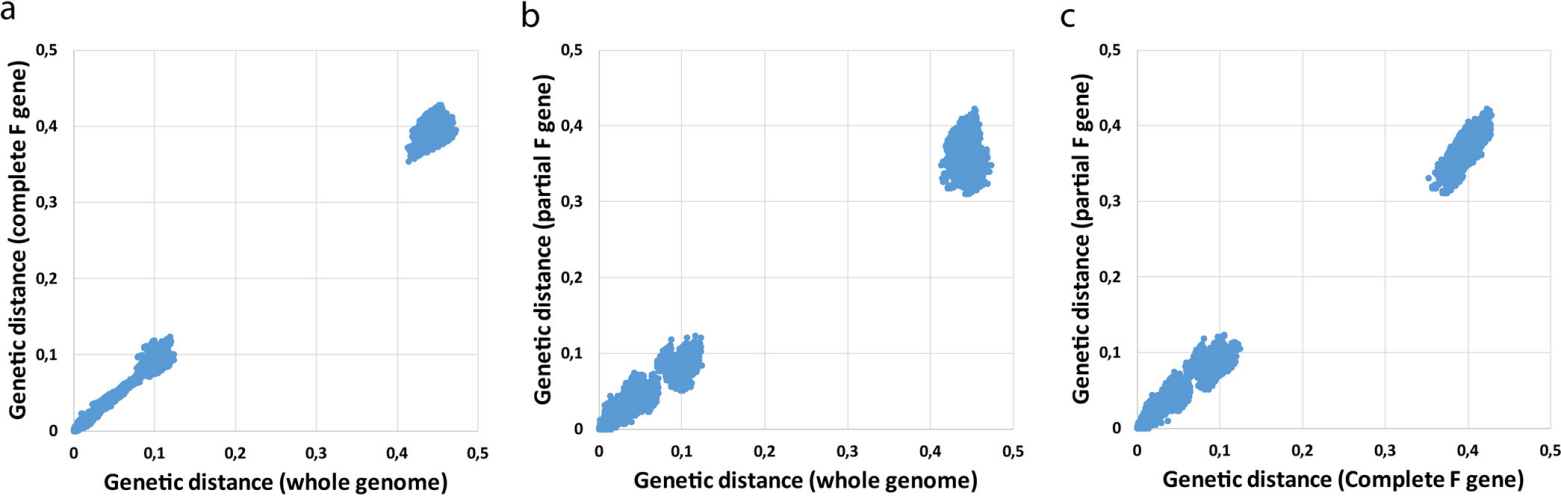
(a to c) Scatterplots of *p* distances between viruses from phylogenetic trees reconstructed from whole-genome sequences versus full-length F gene sequences (a), whole-genome sequences versus partial F gene sequences (b), and full-length F gene sequences versus partial F gene sequences (c). The trees from which *p* distances were compared are shown in Fig. S1, S2, and S3.

The majority of reported phylogenetic analyses have been based on either full-length or partial F gene sequences (in general nucleotide 778 to nucleotide 1226 of the F gene [10]). To compare clustering of viruses based on full-length genome sequences with that based on full-length F or partial F gene sequences, phylogenetic trees were reconstructed from nucleotide alignments of full-length F gene and partial F gene sequences (nucleotide 778 to nucleotide 1226 of the F gene) derived from the 143 full genome sequences described above. No difference in clustering of the viruses was observed between the three analyses, which demonstrates the consistency of lineage topology (Fig. S1, S2, and S3). Interestingly, viruses with a duplication in the G gene also clustered together based on full-length or partial F gene sequences, demonstrating that full-length or partial F gene sequences by themselves carry sufficient information to accurately genotype a virus. To assess whether the phylogenetic distances between viruses were similar in each of the three trees, scatterplots were generated from the *p* distances (the proportion of nucleotide sites at which sequences differ) between all viruses from one tree plotted against those from another tree. The variation in *p* distance between the trees reconstructed from whole-genome sequences and full-length F gene sequences was the smallest (Fig. 2a), while the variation in *p* distances between trees reconstructed from partial F gene sequences and either full-length F gene sequences or whole-genome sequences was slightly larger (Fig. 2b and c), indicating that full-length F gene sequences can be used for phylogenetic analysis with similar accuracy as whole-genome sequences.

10.1128/mbio.02280-22.2 FIG S2 Maximum likelihood phylogenetic tree based on 143 full-length HMPV F gene sequences obtained in this study combined with 56 from GenBank (total *n* = 199). The sequence set is the same as shown in Fig. S1. The tree was reconstructed using the GTR +G +I substitution model with 1,000 bootstraps. Markers on the right indicate virus lineages and sublineages. Download FIG S2, TIF file, 1.9 MB. Copyright © 2022 Groen et al. 2022 Groen et al. https://creativecommons.org/licenses/by/4.0/ This content is distributed under the terms of the Creative Commons Attribution 4.0 International license.

10.1128/mbio.02280-22.3 FIG S3 Maximum likelihood phylogenetic tree based on 143 partial HMPV F gene sequences obtained in this study combined with 56 from GenBank (total *n* = 199). The sequence set is the same as shown in Fig. S1 and S2. The tree was reconstructed using the GTR +G +I substitution model with 1,000 bootstraps. Markers on the right indicate virus lineages and sublineages. Download FIG S3, TIF file, 2.1 MB. Copyright © 2022 Groen et al. 2022 Groen et al. https://creativecommons.org/licenses/by/4.0/ This content is distributed under the terms of the Creative Commons Attribution 4.0 International license.

### Designation of HMPV lineages.

Although the majority of HMPV evolution studies are based on analysis of F gene sequences, some studies rely on analysis of the more variable G gene, while the SH gene is rarely used for these studies. To compare genetic clustering based on the F, G, and SH genes, an alignment was generated from HMPV whole-genome sequences obtained in this study combined with those available from GenBank (*n* = 199). From this alignment, the F, G, and SH genes were selected and used to analyze genetic clustering. For all three proteins, genetic maps were constructed from the sequence alignments. Genetic maps were generated by multidimensional scaling of genetic distance matrixes, which contain all pairwise numbers of nucleotide substitutions between viruses. Distances in the map, represented by a grid, demonstrate the number of nucleotide substitutions between viruses, with each line of the grid representing 10 substitutions. The genetic maps demonstrated clear clustering into six distinct lineages: A1, A2.1, A2.2.1, A2.2.2, B1, and B2 (Fig. S4). Clustering based on F gene sequences resulted in the highest resolution between these six lineages, which supported the use of F gene sequences for designation of lineages.

10.1128/mbio.02280-22.4 FIG S4 (a to c) Comparison of genetic evolution of HMPV based on the analysis of sequences of the three glycoproteins. The genetic maps were constructed from F gene (a), G gene (b), and SH gene (c) alignments that were generated by trimming an alignment containing whole-genome sequences of viruses isolated from hospitalized individuals combined with those obtained from GenBank. The axes represent the genetic distance, with each square of the grid representing a 10-nucleotide distance. Sequences were color coded by their clustering based on F gene sequences in a. Download FIG S4, TIF file, 0.9 MB. Copyright © 2022 Groen et al. 2022 Groen et al. https://creativecommons.org/licenses/by/4.0/ This content is distributed under the terms of the Creative Commons Attribution 4.0 International license.

**FIG 3:**
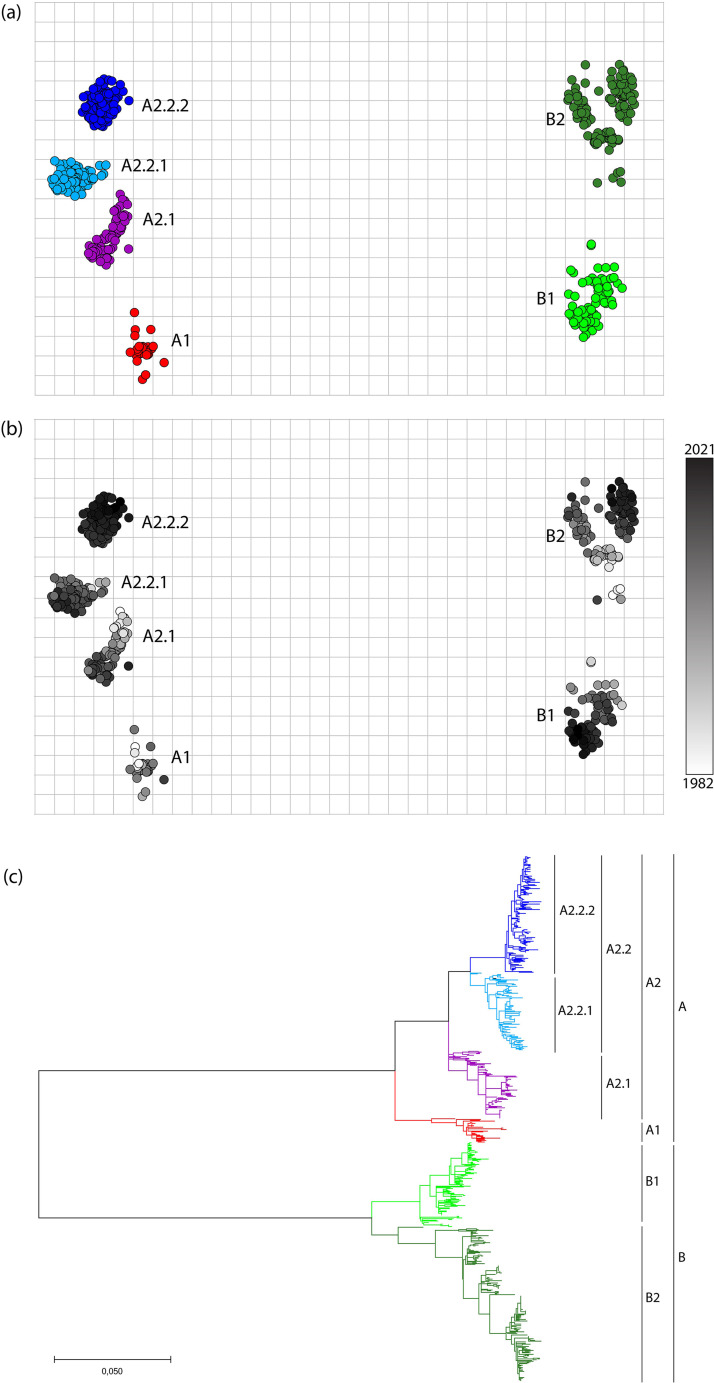
Genetic evolution of HMPV based on full-length F gene sequences. (a and b) A genetic map was generated from full-length F gene sequences obtained in this study combined with those from GenBank (*n* = 744) with color coding per lineage (a) or year of isolation (b). The axes represent genetic distance, with each square of the grid representing a 10-nucleotide distance. Sequences were color coded by year of isolation using a greyscale. (c) Maximum likelihood phylogenetic tree based on 744 full-length HMPV F gene sequences. The tree was reconstructed using the GTR +G +I substitution model with 1,000 bootstraps. The tree is color coded according to the colors used in the map shown in a. The tree with bootstrap values and isolate names is shown in Fig. S5. Markers on the right indicate virus lineages and sublineages.

To propose a robust HMPV classification system, genetic evolution was analyzed based on alignment of full-length F gene sequences obtained in this study combined with those from GenBank (*n* = 744). Analysis of a genetic map and a phylogenetic tree reconstructed from this alignment demonstrated that the viruses clustered in six distinct lineages (Fig. 3a to c; Fig. S5), identical to that described above. The A2.2 lineage displayed gradual evolution over time, while in other lineages, directional evolution was not as clear (Fig. 3b). In the genetic map, there appeared to be a segregation within the B2 lineage, although the border between these two lineages was not clear. This was in agreement with the phylogenetic tree, in which the distance between these two lineages was relatively small (Fig. 3c). To accurately designate the different sublineages, the ranges of nucleotide substitutions between viruses within and between lineages were calculated (Table 2). Overall, the genetic distances within the main lineages A and B were comparable (0 to 129 nucleotide and 0 to 126 nucleotide substitutions between viruses within lineage A and B, respectively). Between lineage A1 and A2, the genetic distance ranged from 68 to 129 nucleotide substitutions. The genetic differences between viruses within the B1 and B2 lineage ranged from 0 to 88 and 0 to 73 nucleotide substitutions, respectively, while between B1 and B2, the genetic distance ranged between 62 and 126 nucleotide substitutions. The genetic distances between viruses within the lineages A2.1, A2.2.1, and A2.2.2 ranged between 0 and 59 nucleotide substitutions, while between A2.1 and the two A2.2 lineages (A2.2.1 and A.2.2.2), this distance was 21 to 75 and 38 to 87 for A2.2.1 and A.2.2.2 lineage viruses, respectively. The distance between viruses from lineages A2.2.1 and A.2.2.2 ranged between 26 and 70 nucleotide substitutions. Although genetic distances within lineage A and B were comparable, two new lineages had evolved within lineage A, while no new lineages have evolved in lineage B.

**TABLE 2:** Nucleotide substitutions within and between all HMPV lineages including the proposed A2.2.1 and A2.2.2 lineages

Lineage	A1	A2.1	A2.2.1	A2.2.2	B1	B2
A1	0–57	68–114	68–122	84–129	234–275	236–264
A2.1		0–59	21–75	39–87	239–278	240–275
A2.2.1			0–45	26–70	247–288	248–284
A2.2.2				0–41	245–281	240–274
B1					0–88	62–126
B2						0–73

10.1128/mbio.02280-22.5 FIG S5 Maximum likelihood phylogenetic tree based on 744 full-length HMPV F gene sequences. The sequence set includes F gene sequences of all 199 full genomes supplemented with 546 additional full-length F gene sequences available from GenBank. The tree was reconstructed using the GTR +G +I substitution model with 1,000 bootstraps. Markers on the right indicate virus lineages and sublineages. Download FIG S5, PDF file, 1.2 MB. Copyright © 2022 Groen et al. 2022 Groen et al. https://creativecommons.org/licenses/by/4.0/ This content is distributed under the terms of the Creative Commons Attribution 4.0 International license.

Based on this analysis, we suggest that the nucleotide substitutions between a newly sequenced virus and viruses from each known lineage can be used to determine to which lineage the new virus belongs. For example, a virus from lineage A2.2.2 should contain between 0 and 41 nucleotide substitutions compared to other lineage A2.2.2 viruses and between 26 and 70 nucleotide substitutions compared to A2.2.1 lineage viruses, etc. If the number of nucleotide substitutions falls within the ranges of each lineage as described in Table 2, the newly sequenced virus can be classified accordingly. Alternatively, if the genetic distance of a newly isolated virus exceeds the ranges of nucleotide substitutions between lineages as described in Table 2, this virus might be considered part of a new lineage. These calculations provide a means to determine which lineage a newly isolated virus belongs to.

### Circulation of virus lineages over time.

**FIG 4:**
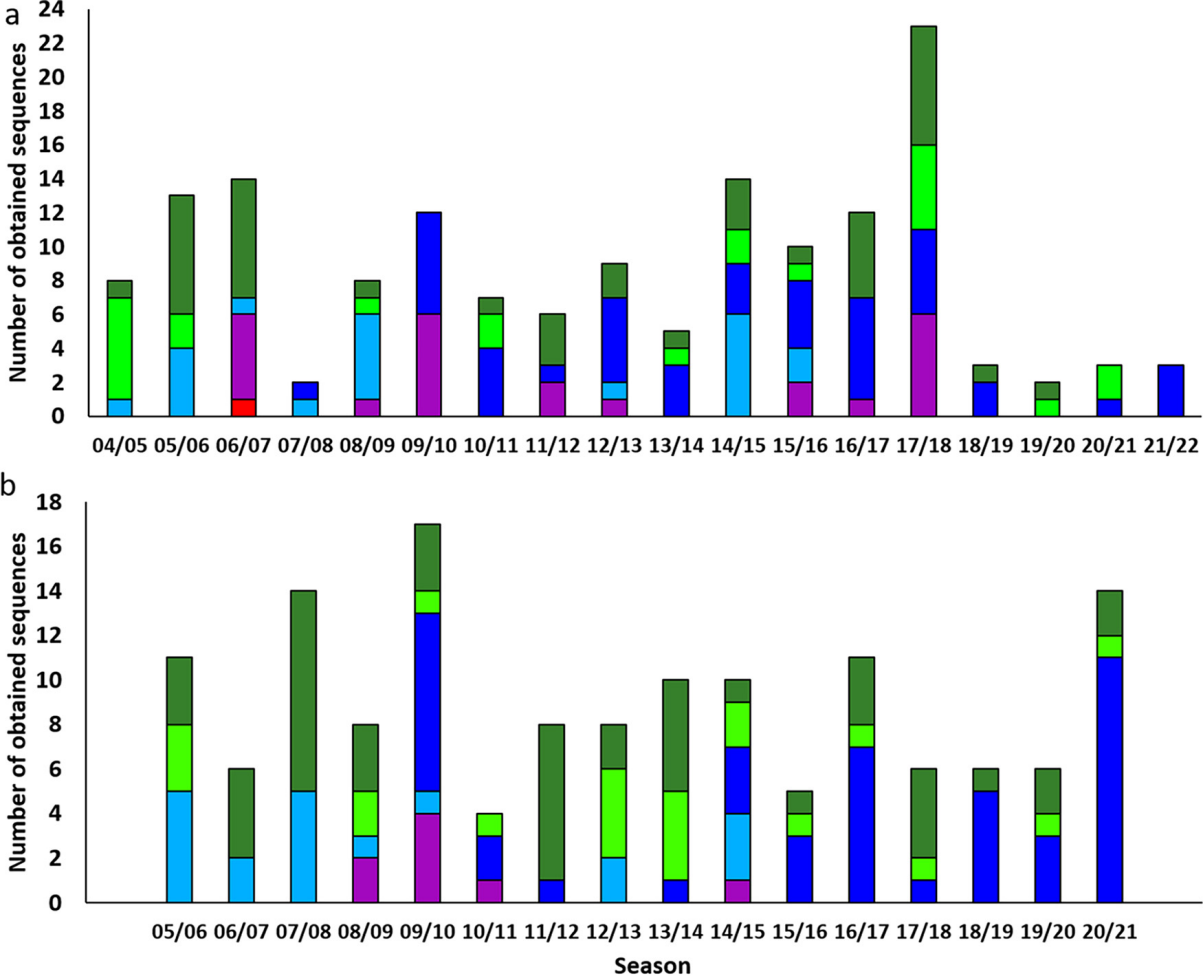
(a) The number of obtained sequences from each lineage of viruses isolated from hospitalized patients suffering from RTIs per epidemiologic season. Seasons started in week 40 and ended in week 39 of the next year. Samples were selected based on a *C_T_* value of ≤25.0, with exceptions as described in the Materials and Methods section, and genotyped by phylogenetic analyses of whole-genome sequences. (b) Clustering of viruses isolated from patients suffering from RTIs visiting general practitioners per year. Samples were selected as described in the Materials and Methods section and genotyped based on phylogenetic analyses of partial F and G gene sequences.

Among viruses obtained from hospitalized individuals between 2005 and 2021, one virus belonged to lineage A1, 15 to lineage A2.1, 21 to lineage A2.2.1, 44 to lineage A2.2.2, 14 to B1, and 35 to B2 (Fig. 4a). The earliest virus of lineage A2.2.2 was obtained in 2008, similar to that described by others (13). The last virus belonging to the A1 lineage was collected in 2006. In this data set, significantly more lineage A viruses (81/130, 62.3%) were detected than lineage B viruses (49/130, 37.7%; χ^2^, *P* = 0.046) (Table 3). It is possible that this is due to the selection bias of samples based on a cutoff quantitative real-time-PCR (qRT-PCR) *C_T_* value of ≤25.0. To investigate whether the underrepresentation of B lineage viruses was due to this *C_T_* cutoff value, 33 additional samples with a *C_T_* value of >25.0 (*C_T_* range of 25.3 to 30.7) were sequenced and genotyped based on lineage-specific amino acid residues of partial F gene sequences (10). Eleven of these viruses (33.3%) belonged to lineage A (1 A1 virus from 2006 and 10 A2 viruses) and 22 (66.6%) to lineage B (8 B1 and 14 B2). Thus, more lineage B viruses were detected in samples with a *C_T_* value of >25.0 than lineage A viruses.

**TABLE 3:** Numbers of viruses belonging to lineage A or lineage B sorted by *C_T_* values (≤25.0 and >25.0) from both hospitalized patients and people visiting general practitioners[Table-fn ngtab3-1]

	*C_T_* ≤ 25.0		*C_T_* > 25.0		Total	
Virus lineage	A	B	A	B	A	B
Hospitalized patients	81/130 (62.3%)	49/130 (37.6%)	11/33 (33.3%)	22/33 (66.7%)	92/163 (56.4%)	71/163 (43.5%)
General practitioners	20/34 (58.8%)	14/34 (41.2%)	52/110 (47.2%)	58/110 (52.8%)	72/144 (50.0%)	72/144 (50.0%)
Total	101/164 (61.6%)	63/164 (38.4%)	62/143 (43.4%)	80/143 (56.6%)	–^*a*^	–

a –, no data.

### Prevalence of HMPV lineages in patients with RTI consulting general practitioners.

**FIG 5:**
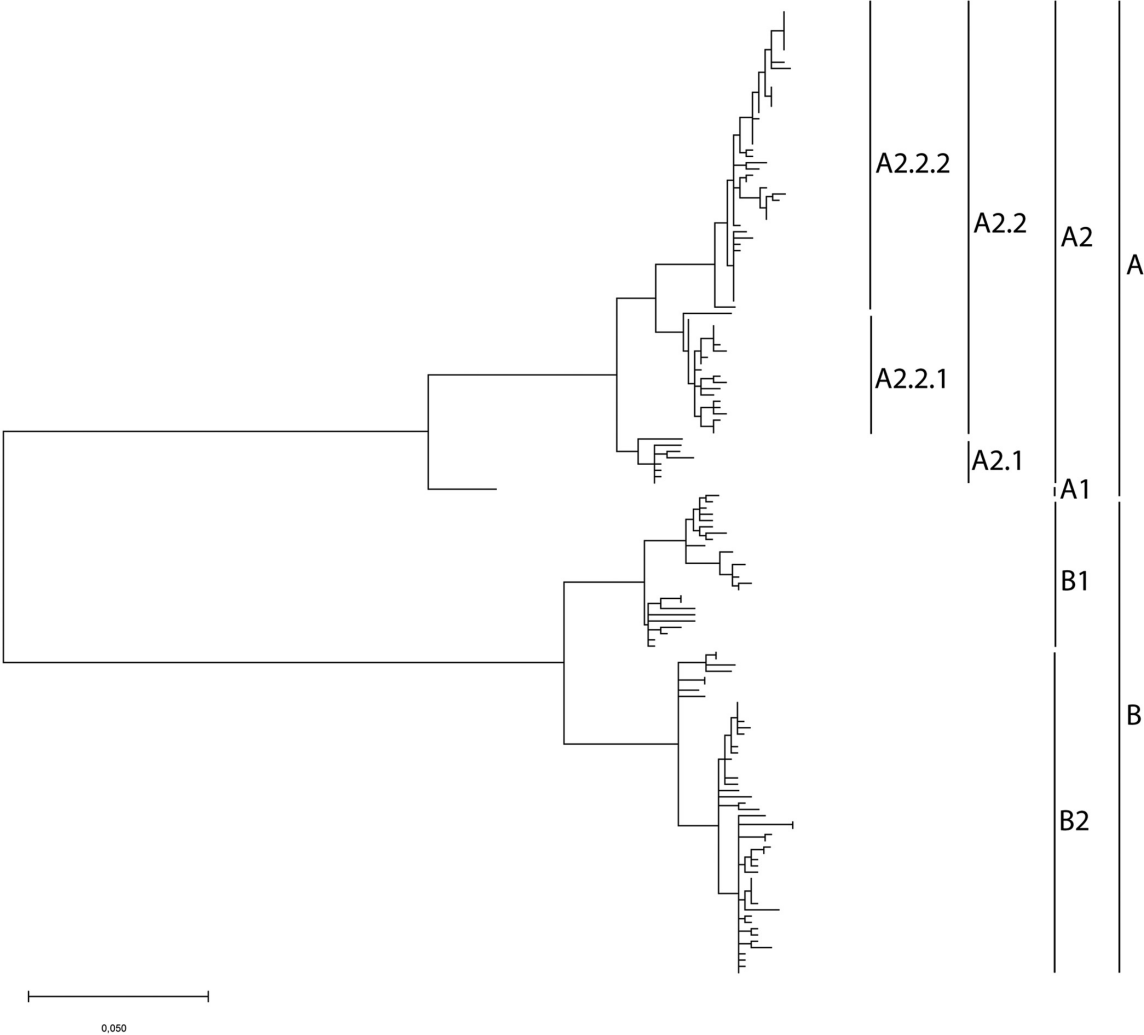
Maximum likelihood phylogenetic tree reconstructed from partial F gene sequences from viruses obtained from patients with RTIs visiting general practitioners combined with those of six viruses obtained from hospitalized individuals. The tree was reconstructed using the GTR +G +I substitution model with 1,000 bootstraps. The tree with bootstrap values and isolate names is shown in Fig. S6. Lines indicate virus lineages (e.g., A2).

To address circulation of HMPV lineages among patients with RTIs consulting general practitioners, 144 viruses collected from samples obtained from patients with RTIs consulting general practitioners in the Netherlands between 2005 and 2021 were genotyped by sequencing of partial F and G genes. The *C_T_* values of these samples determined by qRT-PCR ranged from 18.6 to 38.1. Phylogenetic analysis of these sequences indicated clustering of the viruses in the six lineages similar to the viruses obtained from hospitalized patients (Fig. 4b and 5; Fig. S6). Viruses belonging to lineage A1 were not detected. From the 144 viruses, 71 (49.3%) clustered in lineage A and 73 (50.7%) in lineage B (Table 3). In the samples with *C_T_* values of ≤25.0, the numbers of A and B lineage viruses were not significantly different (20/34 [58.8%] lineage A and 14/34 [41.2%] lineage B, *P* = 0.4651). In samples with a *C_T_* value of >25.0, the numbers of lineage A (52/110, 47.2%) and lineage B viruses (58/110, 52.8%) were not significantly different (*P* ≥ 0.05), although a slightly higher number of lineage B viruses was detected. This contrasted with that of samples isolated from hospitalized patients, in which a significantly higher number of lineage B viruses was detected among samples with a *C_T_* value of >25.0 (Table 3).

10.1128/mbio.02280-22.6 FIG S6 Maximum likelihood phylogenetic tree based on 134 partial HMPV F gene sequences. The sequence set includes 128 partial F gene sequences detected in samples obtained from patients visiting general practitioners and 6 partial F gene sequences from viruses isolated from hospitalized individuals representative of each of the sublineages. The tree was reconstructed using the GTR +G +I substitution model with 1,000 bootstraps. Markers on the right indicate virus lineages and sublineages. Download FIG S6, TIF file, 2.8 MB. Copyright © 2022 Groen et al. 2022 Groen et al. https://creativecommons.org/licenses/by/4.0/ This content is distributed under the terms of the Creative Commons Attribution 4.0 International license.

## DISCUSSION

After the identification of HMPV in 2001, it was reported that worldwide viruses clustered into two genetic lineages that were also antigenically distinct (A and B), which both split into two genetic lineages (A1, A2, B1, and B2) (10). Here, we investigated the evolution of HMPV over the last decades in the Netherlands using phylogenetic analysis of full genome sequences. In this study, phylogenetic analysis of 130 full-length genomes of viruses collected from hospitalized patients with respiratory tract infections between 2005 and 2021 indicated that viruses within our sample set clustered in six genetic lineages: A1, A2.1, A2.2.1, A2.2.2, B1, and B2. Additionally, these data indicate that since 2003, a split in the A2 lineage into lineages A2.1 and A2.2 was observed, and since 2008, lineage A2.2 split into lineages A2.2.1 and A2.2.2. All viruses in lineage A2.2.2 were relatively recent viruses, with the first collected in 2008, and since 2015, this lineage is primarily defined by viruses with a duplication in the G gene. A gradual evolution over time was only clear for lineage A2.2.2 and not so much for the other lineages.

In this study, HMPV evolution based on full genome sequences was studied using viruses circulating in the Netherlands between 2005 and 2021. However, analysis of the F gene sequences of these Dutch isolates together with those circulating worldwide (Fig. 2; Fig. S1 to S3 in the supplemental material) demonstrated that the Dutch strain clustered among those collected worldwide, indicating that the circulation of different genotypes is not specific for the Netherlands.

Viruses clustering in the A2.2.2 lineage with a 111-nucleotide or 180-nucleotide duplication in the G gene have been reported to circulate worldwide since 2014 and 2017, respectively, and the prevalence of viruses carrying a duplication in the G gene has increased over the years (16–18, 20, 28). In our study, although the number of viruses was limited, all A2.2.2 viruses circulating between 2018 and 2021 carried a 111-nucleotide or 180-nucleotide duplication in the G gene.

In line with other studies, no viruses belonging to lineage A1 were detected after 2006. As most qRT-PCR assays used for HMPV diagnostics target the N gene, one explanation for this could be that viruses from this lineage are still circulating but became undetectable due to mutations in the region used in diagnostic assays. More likely, this virus lineage is now extinct or circulating in an area without surveillance.

The samples for the study in hospitalized patients were selected on the basis of *C_T_* values of ≤25 and a maximum of 10 samples per year; therefore, no conclusions can be drawn on the yearly incidence of viruses from the different lineages. However, our study does show that viruses from the different lineages circulate in the same year. Among viruses obtained from hospitalized patients in the Netherlands, a higher number of lineage A than lineage B viruses was detected for most years. This is in contrast to the study among patients with RTIs visiting general practitioners, where lineage A and B viruses were equally often detected. This could be explained by the selection bias of samples from hospitalized patients with a *C_T_* value of ≤25.0, as in samples with a *C_T_* value of >25.0 a higher number of lineage B than lineage A viruses was detected. Additionally, among all samples obtained in this study (from both hospitalized individuals and people visiting general practitioners combined), there was a significantly lower number of B lineage viruses among samples with a *C_T_* value of <25.0 (101/164 [61.6%] lineage A, 63/164 [38.4%] lineage B; *P* = 0.0347). This indicates that, in general, in samples with a *C_T_* value of ≤25.0, lineage A viruses are more often detected than lineage B viruses. Whether this difference in *C_T_* values between lineage A and B viruses corresponds to differences in clinical impact remains to be investigated. Of note, since 2019, diagnostics for detection of HMPV for hospitalized patients has switched from in-house primers and probes to the Panther Fusion System (Hologic), of which the primer and probe sequences are unknown. Primers and probes used to detect HMPV in samples from patients consulting general practitioners were similar to those used to detect HMPV in samples from hospitalized individuals before the introduction of the Panther Fusion System. These differences in assays used for virus diagnostics might result in different sensitivities to detect A or B lineage viruses after 2019.

One of the challenges of comparing HMPV molecular evolution studies is the use of different classification criteria for designating lineages. For other viruses, criteria have been used according to a more unified system, such as for severe acute respiratory syndrome coronavirus 2 (SARS-CoV-2) and influenza virus. For SARS-CoV-2, the Pangolin nomenclature system provides detailed cluster information (29), similar to the Global Initiative on Sharing Avian Influenza Data (GISAID) nomenclature system (https://www.gisaid.org/resources/statements-clarifications/clade-and-lineage-nomenclature-aids-in-genomic-epidemiology-of-active-hcov-19-viruses/), while the Nextstrain nomenclature system offers a more large-scale view of clade trends (https://nextstrain.org/blog/2020-06-02-SARSCoV2-clade-naming). The influenza A(H5) nomenclature system designates new lineages based on monophyletic clusters with bootstrap support values greater than 70% with defined average pairwise *p* distances within and between clades (30). Since 2016, a nomenclature system was proposed for swine influenza A viruses that is in concordance with the nomenclature system for influenza A(H5) viruses (31). While a robust nomenclature system for these viruses is in place, attempts to unify a nomenclature system for RSV have recently started (32). Here, we propose a simple, yet robust, HMPV classification system based on genetic clustering of F gene sequences. The number of nucleotide substitutions of a newly sequenced virus compared to the ranges of nucleotide substitutions within and between the known virus lineages can be used to determine to which lineage a virus belongs. A newly sequenced virus belonging to lineage A2.2.2 should fall within the range of nucleotide substitutions compared to A2.2.1, A2.1, A1, B1, and B2, as described in this study. Alternatively, a virus of which the number of nucleotide substitutions exceeds the ranges of nucleotide substitutions between each of these lineages could be considered part of a new lineage. In addition to this proposed numerical system, to achieve a uniform system for naming new HMPV isolates, we suggest using the guidelines of the International Committee on Taxonomy of Viruses (ICTV) for other viruses, including country codes according to the ISO 3166-1 alpha-2 standards, that is, HMPV/country/isolate number/year/lineage (e.g., HMPV/NL/17/06/A).

The proposed classification system described above supports the split between the A2.2.1 and A2.2.2 lineages. Although a split within lineage B2 seems to be developing, the data thus far do not support this split yet. These findings are in accordance with a study reporting division of the A2.2 lineage into A2.2.1 and A2.2.2 (13), which were referred to as A2b1 and A2b2. However, for HMPV nomenclature, the A and B lineages were initially named as such to define two lineages that were antigenically distinct. The A and B lineages both split into two phylogenetic lineages, which were not antigenically different, named A1, A2, B1, and B2. To prevent confusion about antigenic differences in HMPV nomenclature, we propose to use the nomenclature that follows the guidelines of the ICTV for other viruses, for example, A2.1 instead of A2a and A2.2 instead of A2b (30, 33). There have been reports about the presence of an A2.3 lineage (named A2c in other reports [14]); however, viruses from this proposed lineage cluster within the A2.2.2 lineage in other studies as well as in ours.

Monitoring HMPV evolution is generally done by analyzing sequences of (partial) F or G genes (10, 11, 13, 16, 17, 20, 21, 34). More recently, analyzing whole-genome sequences is being explored as it adds important information compared to partial genome sequences, and the cost-efficiencies of obtaining full genomes by next-generation sequencing have increased considerably (22). Here, we show that phylogenetic clustering based on whole-genome sequences is similar to that of full-length F or partial F gene sequences, although trees reconstructed from partial F gene sequences are slightly more variable.

In conclusion, this study reports on the emergence and potential extinction of genetic HMPV lineages throughout recent decades in the Netherlands. This study suggests that using whole-genome sequences of HMPV captures similar phylogenetic information as the use of full-length F gene sequences. However, new evolutionary events, such as the reported duplication in the G gene, will be missed when only the full-length F gene is analyzed. Finally, phylogenetic analyses were used to propose robust classification criteria for future HMPV lineage designation and virus nomenclature.

## MATERIALS AND METHODS

### Sample selections from hospitalized patients.

HMPV-positive samples obtained from patients hospitalized for RTIs between 2005 and 2021 with a qRT-PCR *C_T_* value of ≤25.0 were selected. For samples obtained before the year 2019, qRT-PCR was performed as described previously (35). For samples obtained after 2019, qRT-PCR was performed using a Panther Fusion System (Hologic), of which the primer and probe sequences are unknown. To obtain an even distribution throughout the years, 10 samples were selected from each year. For seasons with more than 10 samples with a *C_T_* value of ≤25.0, the samples with the lowest *C_T_* values were selected. For seasons with less than 10 samples with a *C_T_* value of ≤25.0, additional samples were selected with a *C_T_* value between 25.0 and 29.0. For some years, either a total number of 10 samples could not be obtained or 10 full-genome sequences could not be obtained. Nine additional samples obtained between 1993 and 2004 were selected based on available materials. Already available full-length genome sequences of prototype viruses for each lineage (e.g., NL/1/00 for lineage A1 and CAN83-97 for lineage A2.1) were also included for references. Patient data were anonymized before receiving the samples, and as a consequence, additional medical ethics review was not needed as consented by the Erasmus MC Medical Ethical Board (MEC-2015-306).

### Selection of samples obtained from patients with RTIs consulting general practitioners.

The general practitioner (GP) sentinel surveillance by the Nivel Netherlands Institute for Health Services Research Primary Care Database and the National Institute for Public Health and the Environment (Rijksinstituut voor Volksgezondheid en milieu; RIVM) reference laboratory (about 40 GP practices spread throughout the Netherlands representing about 0.7% of the Dutch population) collected nasopharyngeal and oropharyngeal swabs combined in one tube of virus transport medium from patients consulting the GP for an RTI (including influenza-like illness). Based on weekly virological records of about 20 clinical diagnostic laboratories in the Netherlands under the auspices of the Dutch Working Group for Clinical Virology (https://www.rivm.nl/virologische-weekstaten), the HMPV epidemic period for each season from 2005 to 2020 was determined. From these periods, a mean of 95 samples (range of 84 to 100) per epidemic period evenly spread over the epidemic period were selected from the GP sentinel surveillance biobank that were negative by qRT-PCR for influenza virus, RSV, rhinovirus, and enterovirus. These samples were subjected to qRT-PCR for HMPV as described previously (10), following nucleic acid isolation by MagNA Pure 96 (Roche) extraction of 200 μL of specimen using a DNA and viral NA small volume kit (Roche). A mean of 10 (range of 4 to 19) samples per epidemic period were found to be positive for HMPV. In 2020/2021, there was no HMPV season due to coronavirus disease 2019 (COVID-19) measures. From January 2021 onward, all collected samples in the GP sentinel surveillance were prospectively tested for HMPV by qRT-PCR. A total of 15 HMPV-positive samples were detected up to October 2021 that were included in the study. Due to lower viral loads in the GP sentinel surveillance patients than in hospitalized patients, all qRT-PCR HMPV-positive specimens irrespective of *C_T_* value (median *C_T_* value of 29; range of 19 to 39) were subjected to partial F gene and G gene PCR and Sanger sequencing. For these samples, a nested PCR was performed to increase efficiency for lower viral load specimens (F PCR: forward primer 5′-TGTTGTGCGGCARTTTTCAGAC-3′ and reverse primers 5′-GCACTGCTTAGGATTCTGTTTGA-3′ and 5′-TGCACTGTTYAGRATTTTGTTTGAC-3′; nested F PCR: forward primers 5′-TGGAATAACACCAGCAATATCCTT-3′ and 5′-AATGCAGGGATAACACCAGCAAT-3′ and reverse primer 5′-CTGTTCRCCYTCAACTTTGCT-3′; G PCR: forward primer 5′-TACAAAACAAGAACATGGGACAAG-3′ and reverse primers 5′-GTTTCACTGAAAGATATTACTCCTTT-3′ and 5′-GTCTCACTAAAAGAAATCACTCCTTT-3′; nested G PCR: forward primers 5′-ATGGAGGTGAAAGTGGAGAACATTC-3′ and 5′-ATGGAAGTAAGAGTGGAGAACATTC-3′ and reverse primer 5′-GAGATAGACATTAACAGTGGATTC-3′). The primers used for Sanger sequencing of PCR products were the same as those used for the nested PCR.

### Virus nomenclature.

Nomenclature of viruses followed the guidelines of the ICTV for other viruses, for example, HMPV/country/isolate number/year/lineage (i.e., HMPV/NL/17/06/A) (33).

### MinION sequencing and PCR of the G gene and partial HMPV F gene.

HMPV-positive samples were used for RNA isolation and MinION sequencing as described previously (27). PCR of the HMPV F gene was performed as described previously (27). The presence or absence of 111-nucleotide and 180-nucleotide duplications was confirmed by Sanger sequencing as described previously (27). Gaps in consensus sequences were resolved by PCR amplification of the missing amplicon followed by Sanger sequencing as described previously (27). Sequencing of partial F genes (nucleotide positions 778 to 1226 of the F open reading frame [ORF]) for genotyping of samples obtained from hospitalized individuals with a *C_T_* value of >25.0 was performed as described previously (10). All full-length F gene sequences available from GenBank were downloaded as a FASTA alignment file.

### Phylogenetic analysis.

Nucleotide sequence alignments were generated using MAFFT (https://mafft.cbrc.jp/alignment/server/). Maximum likelihood phylogenetic analyses were conducted using the MEGA-X software with the best fit DNA model as determined by the MEGA-X software with 1,000 bootstraps. The best fit model for each phylogenetic tree is described in the figure legends. Pairwise distances were generated by MEGA-X software.

### Clustering of HMPV in genetic maps.

To visualize the clustering of HMPV sequences based on the number of nucleotide substitutions between them, genetic maps representing HMPV nucleotide sequences were generated essentially as described previously (36, 37). Nucleotide sequence alignments were generated for various HMPV open reading frames using MAFFT, and a genetic distance matrix was generated with all pairwise numbers of nucleotide substitutions. A multidimensional scaling algorithm was used to generate two-dimensional representations of the matrices with Euclidian distances (36).

### Data availability.

All full genome sequences from viruses isolated from hospitalized individuals generated in this study were submitted to GenBank with accession numbers OL794355 to OL794483. Partial F and G gene sequences form viruses isolated from patients visiting general practitioners were submitted to GenBank with accession numbers ON461481 to ON461747.
