# Supplementary material for: Emergence and Potential Extinction of Genetic Lineages of Human Metapneumovirus between 2005 and 2021
Source: mBio. 2022 Dec 12;14(1):e02280-22. doi: 10.1128/mbio.02280-22 (PMC9973309; doi:10.1128/mbio.02280-22)
Supplement: TABLE S1 [file mbio.02280-22-s0002.pdf]

Table S1. Accession numbers of viruses used as reference for phylogenetic analysis of samples obtained from hospitalized individuals (Figure 1).

Reference virus	Origin
**AB503857.1**	GenBank
**AY297748.1**	GenBank
**MK820375.1**	GenBank
**AY297749.1**	GenBank
**EF535506.1**	GenBank
**GQ153651.1**	GenBank
**HM197719.1**	GenBank
**MK588633.1**	GenBank
**MK588635.1**	GenBank
**JN184399.1**	GenBank
**KC403972.1**	GenBank
**KC403973.1**	GenBank
**KC403974.1**	GenBank
**KC403976.1**	GenBank
**KC403984.1**	GenBank
**KC562219.1**	GenBank
**KC562220.1**	GenBank
**KC562221.1**	GenBank
**KC562222.1**	GenBank
**KC562223.1**	GenBank
**KC562232.1**	GenBank
**KC562234.1**	GenBank
**KC562235.1**	GenBank
**KC562238.1**	GenBank
**KC562239.1**	GenBank
**KC562241.1**	GenBank
**KC562244.1**	GenBank
**KF516922.1**	GenBank
**KF530173.1**	GenBank
**KF530179.1**	GenBank
**KJ627383.1**	GenBank
**KJ627392.1**	GenBank
**KJ627401.1**	GenBank
**KJ627414.1**	GenBank
**KJ627417.1**	GenBank
**KJ627419.1**	GenBank
**KJ627430.1**	GenBank
**KJ627432.1**	GenBank
**KJ627430.1**	**GenBank**
**KJ627432.1**	GenBank
**KJ627433.1**	GenBank
**KJ627435.1**	GenBank
**KM361520.1**	GenBank
**KU821121.1**	GenBank
**KY474537.1**	GenBank
**MK087726.1**	GenBank
**MK167039.1**	GenBank
**MK167040.1**	GenBank
**MK588635.1**	GenBank
**MK588636.1**	GenBank
**MK588637.1**	GenBank
**MK820375.1**	GenBank
**MN306019.1**	GenBank
**MN306028.1**	GenBank
**MN745086.1**	GenBank
**MN745087.1**	GenBank
**HMPV_Yokohama_P_8945_2017**	Nao et al. 2020
**HMPV_Yokohama_P_9299_2018**	Nao et al. 2020
**AF371337.2**	Reference NL/1/00/A1
**FJ168779**	Reference NL/1/94/B2
**AY525843**	Reference NL/1/99/B1
**FJ168778**	Reference NL/17/00/A2
