# Supplementary figures and images for: Emergence and Potential Extinction of Genetic Lineages of Human Metapneumovirus between 2005 and 2021
Source: mBio. 2022 Dec 12;14(1):e02280-22. doi: 10.1128/mbio.02280-22 (PMC9973309; doi:10.1128/mbio.02280-22)

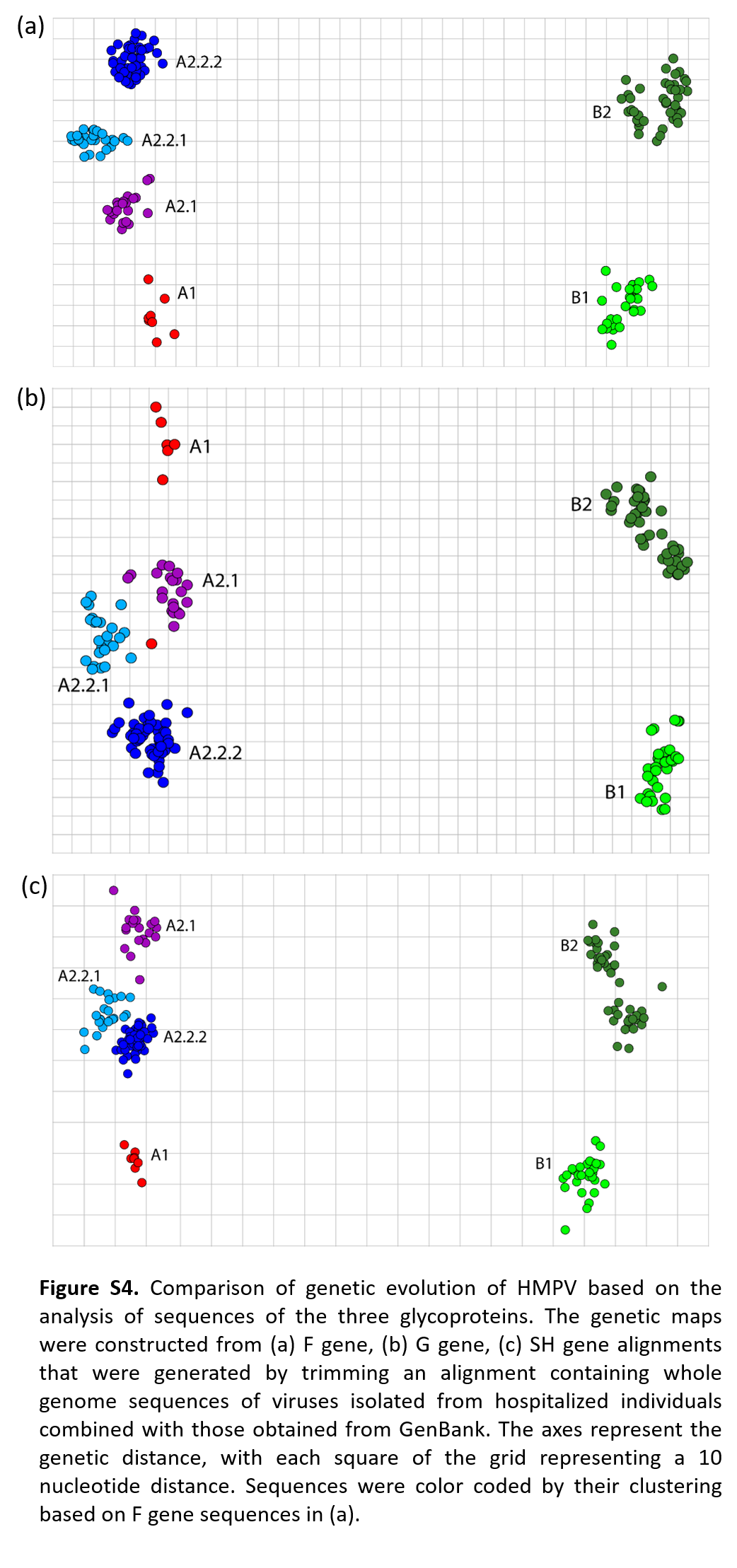

Supplement: FIG S4 [file mbio.02280-22-s0005.tif]
