# Supplementary material for: Emergence and Potential Extinction of Genetic Lineages of Human Metapneumovirus between 2005 and 2021
Source: mBio. 2022 Dec 12;14(1):e02280-22. doi: 10.1128/mbio.02280-22 (PMC9973309; doi:10.1128/mbio.02280-22)
Supplement: FIG S5 [file mbio.02280-22-s0006.pdf]

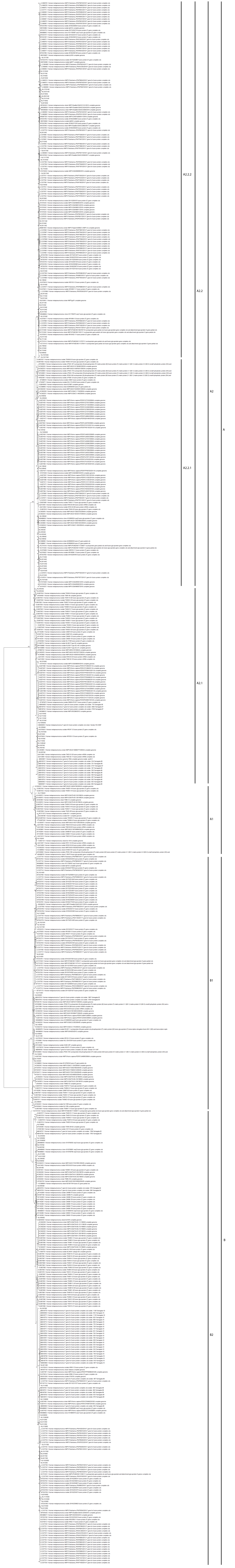

Figure A5. Maximum likelihood phylogenetic tree based on 744 full-length HMPV F gene sequences. The sequence set includes F gene sequences of all 199 full genomes supplemented with 546 additional full-length F gene sequences available from GenBank. The tree was reconstructed using the GTR +G +I substitution model with 1000 bootstraps. Markers on the right indicate virus lineages and sublineages.
